# Mitophagy Upregulation Occurs Early in the Neurodegenerative Process Mediated by α-Synuclein

**DOI:** 10.1007/s12035-024-04131-6

**Published:** 2024-04-06

**Authors:** Sarah Hui, Jimmy George, Minesh Kapadia, Hien Chau, Zahn Bariring, Rebecca Earnshaw, Kashfia Shafiq, Lorraine V. Kalia, Suneil K. Kalia

**Affiliations:** 1 Krembil Research Institute, Toronto Western Hospital, University Health Network, Toronto, ON Canada; 2 Division of Neurology, Department of Medicine, University of Toronto, Toronto, ON Canada; 3 Tanz Centre for Research in Neurodegenerative Diseases, University of Toronto, Toronto, ON Canada; 4 Division of Neurosurgery, Department of Surgery, University of Toronto, Toronto, ON Canada; 5 KITE, University Health Network, Toronto, ON Canada; 6 CRANIA, Toronto, ON Canada

**Keywords:** Parkinson’s disease, Alpha-synuclein, Mitochondria dysfunction, Mitophagy, Lysosomes, Neurodegeneration, Substantia nigra pars compacta

## Abstract

Parkinson’s disease (PD) is a progressive neurogenerative movement disorder characterized by dopaminergic cell death within the substantia nigra pars compacta (SNpc) due to the aggregation-prone protein α-synuclein. Accumulation of α-synuclein is implicated in mitochondrial dysfunction and disruption of the autophagic turnover of mitochondria, or mitophagy, which is an essential quality control mechanism proposed to preserve mitochondrial fidelity in response to aging and stress. Yet, the precise relationship between α-synuclein accumulation, mitochondrial autophagy, and dopaminergic cell loss remains unresolved. Here, we determine the kinetics of α-synuclein overexpression and mitophagy using the pH-sensitive fluorescent mito-QC reporter. We find that overexpression of mutant A53T α-synuclein in either human SH-SY5Y cells or rat primary cortical neurons induces mitophagy. Moreover, the accumulation of mutant A53T α-synuclein in the SNpc of rats results in mitophagy dysregulation that precedes the onset of dopaminergic neurodegeneration. This study reveals a role for mutant A53T α-synuclein in inducing mitochondrial dysfunction, which may be an early event contributing to neurodegeneration.

**Supplementary Information:**

The online version contains supplementary material available at 10.1007/s12035-024-04131-6.

## Introduction

Parkinson’s disease (PD) is a complex neurogenerative disease characterized by progressive loss of dopaminergic neurons within the substantia nigra pars compacta (SNpc) and intraneuronal inclusions known as Lewy pathology which contain aggregates of the protein α-synuclein [1, 2]. The exact cause of neuronal death in PD is not fully understood, but multiple lines of evidence point to the accumulation of α-synuclein as an upstream event. For example, inherited forms of PD can be caused by missense mutations (e.g., A53T) or multiplications in the *SNCA* gene which encodes for α-synuclein [3]*.* There is also mounting evidence that supports a central role for mitochondrial dysfunction in the neurodegenerative process as it is a feature observed in both idiopathic PD [4] and rare inherited forms, such as autosomal recessive familial PD [5]. Interestingly, α-synuclein can localize within the mitochondria as it contains a cryptic mitochondrial targeting sequence [6, 7], and it has an affinity for mitochondria-specific cardiolipin [7] which can induce rapid aggregation of A53T α-synuclein [8]. Mutant α-synuclein increases the production of reactive oxygen species (ROS) [6], drives mitochondria fragmentation [9, 10], and impairs complex I function [6, 11]. Furthermore, α-synuclein can also impair and/or induce mitochondrial autophagy, or mitophagy [11–16].

Mitophagy is an essential process for maintaining cellular homeostasis in which aged or damaged mitochondria are selectively removed and degraded via the lysosomal system [17]. Dysregulation of mitophagy has been implicated in neurodegenerative diseases, including PD, [18, 19], and its restoration may be a potential therapeutic strategy to mitigate neurodegeneration [20]. Although recent data suggests that α-synuclein can alter mitophagy levels [11–16], there is no clear consensus as to how it impacts this process, and the use of different mitophagy reporters with various model systems limits interpretation and comparison of findings across studies. For example, the application of extracellular α-synuclein aggregates to rat pheochromocytoma (PC12) cells caused a reduction in mitochondrial protein ubiquitination and mitochondria-containing autophagosomes suggesting a reduction in mitophagy [14], whereas overexpression of A53T α-synuclein in primary rat cortical neurons resulted in increased co-localization of the EGFP-LC3 autophagosome and mitochondrial-pDSRed2 markers which was interpreted as an increase in mitophagy [12]. Furthermore, there are few in vivo studies examining the temporal relationship between α-synuclein accumulation, mitophagy, and dopaminergic neurodegeneration [11, 13]. Here, we define this relationship by using the mito-QC reporter, a pH-sensitive dual fluorophore that localizes to the outer mitochondrial membrane (OMM) and produces easily quantifiable mCherry puncta-like structures when mitochondria are engulfed within lysosomes [21], to examine mitophagy due to A53T α-synuclein accumulation in vitro and in vivo.

## Methods

### SH-SY5Y Cell Culture Plasmid Transfection and Bafilomycin Treatment

SH-SY5Y cells were cultured in Dulbecco’s modified Eagle medium (DMEM)/F12 medium (Sigma) supplemented with 10% (v/v) fetal bovine serum (Life Technologies) and 1% antibiotic–antimycotic (Life Technologies). The cells were maintained at 37 °C in a humidified atmosphere of 5% (v/v) CO_2_. Cells were grown to 50% confluence in 24-well culture dishes and then transfected with plasmids expressing the mito-QC reporter, wild-type (WT) α-synuclein, mutant A53T α-synuclein, blue fluorescent protein (BFP), and pcDNA control using Lipofectamine 2000 (Thermo Fisher Scientific) according to the manufacturer’s protocol. For experiments on lysosomal function, transfected cells were treated with bafilomycin A1 (40 nm) or DMSO (vehicle) for 6 h.

### Adeno-Associated Viruses

Adeno-associated virus (AAV) of a 1/2 serotype, under the CAG promotor, a hybrid of chicken beta actin (CBA) promotor fused with the cytomegalovirus (CMV) immediate early enhancer sequence, was used to express human A53T α-synuclein (AAV-A53T) (2.55 × 10^12^ genomic particles (gp) per mL; GeneDetect Ltd) as previously described [22]. An AAV 1/2 vector expressing α-synuclein with a scrambled sequence (AAV-Scr) was used as a control for in vitro experiments in neuronal cultures. The scrambled and non-scrambled human A53T α-synuclein were tagged with hemagglutinin (HA) on the carboxy-terminal end. The scrambled sequence has previously been used [23]. An AAV1/2 lacking the A53T α-synuclein open reading frame was used as an empty vector control (AAV-Empty) for in vivo experiments in mice. The mCherry-EGFP-FIS1 (mito-QC) open reading frame was expressed as an AAV serotype 8 under the control of the CAG promotor (2.18 × 10^12^ gp; Vector Builder Inc.). Mito-QC reporter is a pH-sensitive dual fluorophore reporter that localizes to the OMM and can be utilized to measure levels of mitophagy [21].

### Primary Cortical Neuron Culture and AAV Transduction

Primary cortical neurons were prepared from embryonic day 17 (E17) Sprague–Dawley rats purchased from Charles River. Embryos were surgically separated from the mothers, and the cortices were dissected in Hank’s balanced saline solution (Gibco). Following the removal of the meninges, using the papain dissociation system (Worthington), cells were dissociated and subsequently cultured in a Neurobasal medium supplemented with GlutaMax™ (Gibco), factor B27 (Gibco), and antibiotic–antimycotic (Gibco). Cells were plated on 24-well plates coated with a poly-D-Lysine solution at 37 °C in 5% CO_2_, with half media changes every 2 days. Cells were transduced with AAV-A53T/AAV-Scr and AAV-mito-QC 2 days post-isolation at a multiplicity of infection (MOI) of 3000 and 1300, respectively. Virus was removed 72 h after transduction by a complete media change, and cells were collected 24 h later.

### Immunofluorescence Staining of Cultured Cells

Post-fixation, cells were permeabilized with 0.2% Triton X-100 for 15 min followed by overnight incubation of primary antibodies diluted in PBS at 4 °C. Primary antibodies used include anti-α-synuclein (Invitrogen; 211, 32–8100), anti-LC3B (Cell Signaling Technologies; 3868S), anti-TOMM20 (Abcam; EPR15581-39), and anti-HA (Roche; 3F10, ROAHAHA). Primary antibodies were washed off with PBS, and cells were incubated for 1 h at room temperature with secondary antibodies diluted in PBS. Secondary antibodies were washed off, and nuclei were counterstained with DAPI (Thermo Fisher) prior to coverslips being mounted on slides using a fluorescent mounting medium (DAKO).

### Western Blot Analysis

Cells were harvested and lysed using RIPA buffer with EDTA-free protease inhibitor cocktail and phosSTOP phosphatase inhibitor cocktail (Roche). 6X sodium dodecyl-sulfate polyacrylamide gel electrophoresis (SDS-PAGE) sample buffer was added to the whole cell lysate and boiled for 10 min at 95 °C. Samples were subsequently run on a 10% SDS-PAGE gel. Gel was transferred onto a polyvinylidene fluoride membrane (Bio-Rad) and blocked at room temperature in 5% w/v milk in 0.1% TBS-T for 1 h. The membrane was probed with primary antibodies overnight at 4 °C: anti-GFP (Cell Signaling Technologies; 2956), anti-α-synuclein (Invitrogen; 211 32–8100), and anti-GAPDH (Cell Signaling Technologies; 2118S). Immunoblots were developed using HRP-conjugated secondary antibodies and chemiluminescent detection methods.

### Animals

Ten-week-old adult female Sprague–Dawley rats (230–280 g) were purchased from Charles River. Animals were pair-housed in cages with wood bedding with access to food and water ad libitum and were maintained on a consistent 12-h light/dark cycle. The University Health Network Animal Care Committee approved all animal procedures in accordance with the guidelines and regulations set by the Canadian Council on Animal Care.

### Stereotaxic Surgery

During the surgical procedure, animals were secured in a stereotaxic frame and anesthetized under isoflurane/oxygen anesthesia. Anafen (5 mg/kg) was subcutaneously administered as an analgesic. Prior to incision, the surgical site was shaved and sterilized with an iodine/betadine solution. Unilateral co-injections of the viruses targeting the SNpc on the right side of the brain were performed using the coordinates AP 5.2 mm, ML 2 mm, and DV 7.5 mm with respect to bregma. Two microliters of viral vector at a final titer of 2.8 × 10^12^ genomic particles/mL was injected at a rate of 0.5 μ/min using a 10-μL Hamilton syringe with a 26-gauge needle as previously described [24–26]. Post-viral injection, the needle remained in place for 5 min.

### Tissue Preparation

Animals were euthanized by cardiac puncture under isoflurane/oxygen anesthesia, followed by transcardial perfusion with heparinized saline. To isolate the brain, rats were decapitated using a guillotine. The anterior portion of the brain was flash frozen in 2-methylbutane (Sigma) and stored at − 80 °C. One-millimeter-thick sections of the ventral striatum were cut using a matrix and frozen at − 80 °C. The posterior portion of the brain (posterior striatum and SN) was submerged in 4% PFA in 0.1 M PBS for 48 h, followed by 15% sucrose in PBS and 30% sucrose in PBS for cryoprotection. Using a sliding microtome (Leica Microsystem Inc.), 40-μm coronal cryosections were collected in a series of six sections and stored in anti-freeze media (30% glycerol, 30% 2-ethoxyethanol, 40% PBS) at − 20 °C.

### Immunofluorescence Staining of Cryosections

Free-floating brain sections were washed with phosphate-buffered saline (PBS) and 0.2% Tween 20 (0.2% PBS-T). Sections were blocked in blocking buffer (10% normal goat serum (NGS), 2% BSA in 0.2% PBS-T) for 1 h at room temperature. Following blocking, sections were incubated with primary antibodies diluted in 2% NGS in 0.2% PBS-T overnight at room temperature. Primary antibodies were washed off with 0.2% PBS-T, and secondary antibodies were diluted in 2% NGS in 0.2% PBS-T and incubated at room temperature for 1 h (Online Resource 1). Secondary antibodies were washed off with 0.2% PBS-T, and sections were mounted on slides using a fluorescent mounting medium (DAKO).

### Image Acquisition and Analysis

Confocal images were captured by a Zeiss LSM880 confocal microscope. Both SH-SY5Y cells and rat primary cortical neurons were imaged at 63 × magnification with 405-nm, 488-nm, 555-nm, and 639-nm laser lines. For SH-SY5Y and primary cortical neurons, Z-stacks were taken within linear range at a constant gain for each channel at a 1024 × 1024 and 3000 × 3000-pixel resolution respectively. For immunohistochemistry, the whole SNpc was imaged at 10 × magnification with 405-nm, 488-nm, 555-nm, and 639-nm laser lines. Images were taken at a constant gain for each channel at a 1432 × 972-pixel resolution. Six serial coronal sections were imaged per animal, separated by 240-μm intervals. Approximately four consecutive coronal sections were imaged per animal.

For quantification of the number of mito-lysosomes per cell, contours were manually drawn around the neuron body and processes to define the region of interest (ROI). Using the IMARIS, spots module puncta was selected in both the mCherry and EGFP channels at an approximate size of 0.4-μm diameter. The “co-localize spots” function was used to measure the number of mCherry puncta that were not co-localized with EGFP puncta. For quantification of the percentage of LC3B co-localized with TOMM20, contours were manually drawn around cells to define the region of interest (ROI). Using IMARIS, the spots module was used to identify LC3B and the surfaces module was used to identify TOMM20. The “spots close to surface” function was used to measure the number of LC3B puncta that were co-localized with TOMM20 and the number that were not. The percentage of LC3B co-localized with TOMM20 was calculated by dividing the number of co-localized LC3B spots by the total number of LC3B spots. To analyze SNpc images via HALO, contours were manually drawn around the ipsilateral SNpc to define the ROI as we have previously described [27]. Real-time modifications were created to determine analysis parameters for the automated detection of the signal of interest. For quantification of the mito-QC signal, the co-localization module was used to measure the red and green signals of the cells within the ROI. To measure mitophagy in the SNpc, the red-only signal was quantified (red fluorescence signal not co-localized with green fluorescent signal). To detect DA neurons, cells were counted by measuring TH + immunofluorescent-labeled cells within the ROI. Quantification of DA neurons is represented as total cells divided by the total analyzed area.

### Data Analysis

For in vitro studies, three independent experiments were conducted for each, and a minimum of eight cells were analyzed per experiment. For in vivo studies, five to six animals per group were analyzed for the week 1 timepoint and nine to ten animals for the week 3 timepoint. Data are presented as mean ± s.e.m. GraphPad Prism version 8 software (GraphPad Software Inc.) was used to perform all data analysis, and the specific statistical tests performed are indicated.

## Results

### Accumulation of Mutant α-Synuclein Is Associated with Increased Mitophagy in SH-SY5Y Cells

**Fig. 1:**
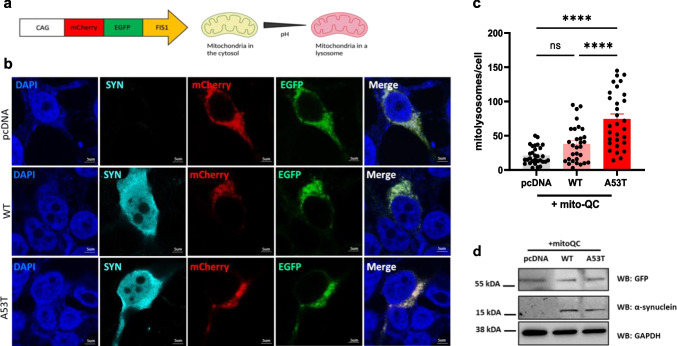
Mutant α-synuclein overexpression leads to increased levels of mitophagy in SH-SY5Y cells. **a** Schematic depicting the mito-QC mitophagy reporter [21]. When mito-QC co-localizes to the outer mitochondrial membrane, it fluoresces both green and red; however, when mitochondria are engulfed in a lysosome, the acidic environment quenches the green signal and results in the mCherry-only punctate-like fluorescence. **b** Representative confocal images of SH-SY5Y cells transfected with mito-QC in combination with human wild-type (WT) α-synuclein, mutant A53T α-synuclein, or pcDNA control. Nuclear counter staining with DAPI (blue), anti-α-synuclein (cyan), mCherry (red), and EGFP (green). Scale bar 5 μm. **c** SH-SY5Y cells expressing mutant A53T α-synuclein exhibit an increase in the number of mito-lysosomes (74.3 ± 7.4) in comparison to cells overexpressing WT α-synuclein (37.9 ± 4.9) or pcDNA control (21.1 ± 2.35) (one-way ANOVA, *F*(2,87) = 26.20; *P* < 0.0001; followed by Tukey’s m.c.t., *n* = 3 independent experiments, analyzing 10 cells per condition). No significant difference in the number of mito-lysosomes was observed between WT α-synuclein and pcDNA control conditions. Data in the graph represents means ± s.e.m. ****P < 0.0001; ns indicates *P* > 0.05. **d** Representative Western blot demonstrating comparable overexpression of WT α-synuclein and mutant A53T α-synuclein in the context of the mito-QC reporter in SH-SY5Y cell lysates harvested 24 h post-transfection

To determine the effects of α-synuclein accumulation on mitophagy, we first co-expressed the pH-sensitive mito-QC mitophagy reporter (Fig. 1a) with human WT α-synuclein, A53T α-synuclein, or pcDNA control in human SH-SY5Y cells, an immortalized cell line with dopaminergic features [28]*.* Immunofluorescence analysis 24 h post-transfection revealed that WT α-synuclein overexpression did not alter the frequency of mCherry-only punctate-like fluorescence, indicative of mito-lysosomes, when compared to the pcDNA control condition (Fig. 1b, c). However, overexpression of mutant A53T α-synuclein resulted in an elevated number of mito-lysosomes per cell compared to both WT α-synuclein and pcDNA control conditions (Fig. 1b, c). Western blot analysis of cell lysates confirmed comparable overexpression of WT α-synuclein and mutant A53T α-synuclein in the context of the mito-QC reporter in SH-SY5Y cells (Fig. 1d). To validate the specificity of this response, we first overexpressed blue fluorescent protein (BFP) with the mito-QC reporter and demonstrated that BFP-transfected cells do not exhibit a rise in mito-lysosomes when compared to pcDNA-transfected control cells (Fig. S1a–b). Next, we confirmed the effect of mutant A53T α-synuclein on mitochondrial turnover by analyzing the co-localization between LC3-positive autophagosomes and TOMM20-labeled mitochondria in transfected SH-SY5Y cells. In line with our findings with the mito-QC reporter, overexpression of mutant A53T α-synuclein, but not WT α-synuclein, led to an increase in the number of LC3-positive autophagosomes that co-localized with TOMM20-labeled mitochondria in comparison to pcDNA-transfected cells (Fig. S2a–b). Taken together, our results suggest that the accumulation of mutant α-synuclein promotes mitophagy in a PD-relevant cell model.

### Accumulation of Mutant α-Synuclein Is Associated with Increased Mitophagy in Neurons

**Fig. 2:**
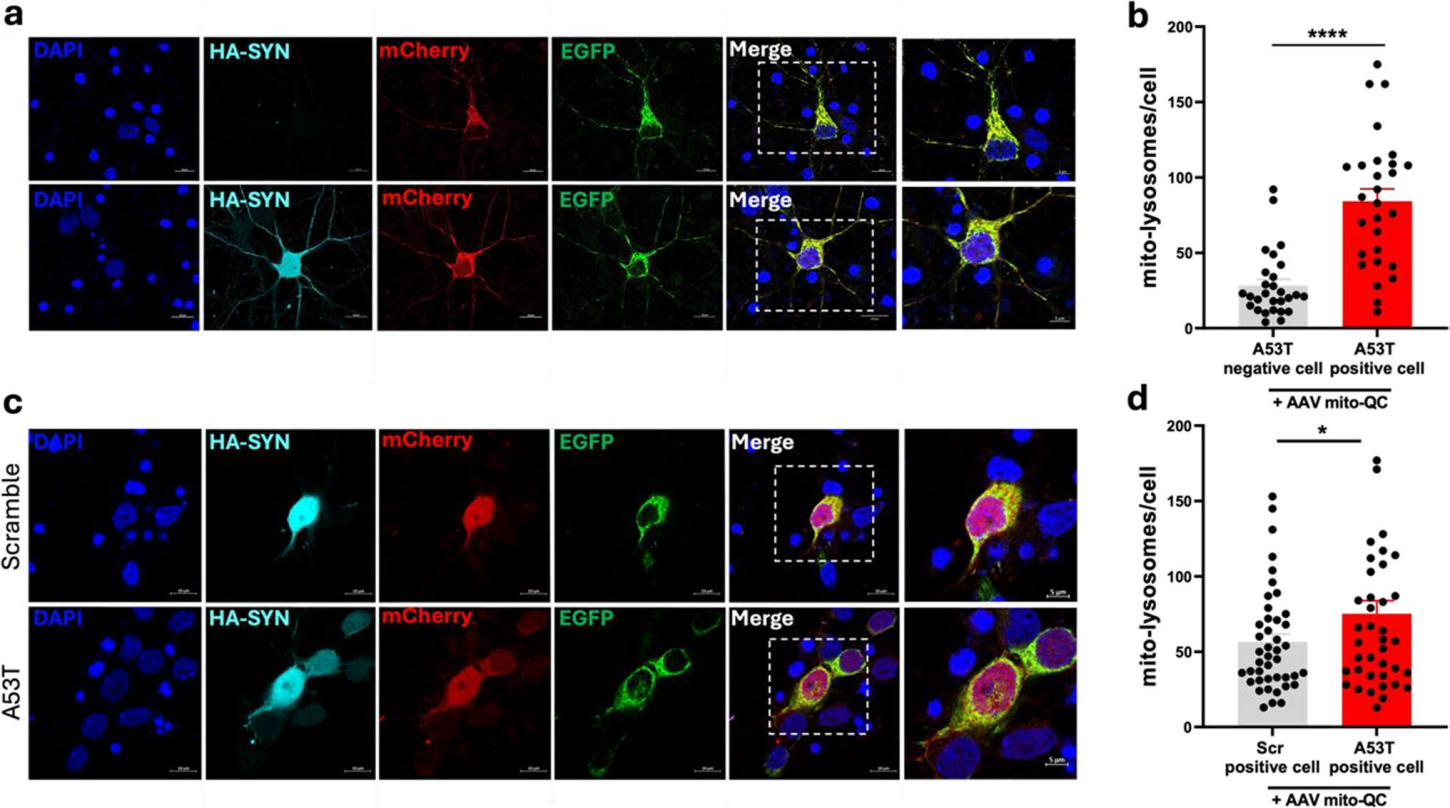
Mutant α-synuclein overexpression leads to increased levels of mitophagy in rat primary cortical neurons. **a** Representative confocal images of primary rat cortical neurons infected with adeno-associated viruses expressing mito-QC (AAV-mito-QC) and HA-tagged A53T α-synuclein (AAV-A53T). Nuclear counter staining with DAPI (blue), anti-α-synuclein (cyan), mCherry (red), and EGFP (green). Scale bar 10 μm (inset 5 μm). **b** Primary rat cortical neurons expressing A53T α-synuclein display an increase in mito-lysosomes (84.09 ± 4.08) in comparison to neurons negative for A53T α-synuclein (28.18 ± 8.25) (two-tailed unpaired *T* test, *t*(54) = 6.07; *P* < 0.0001; *n* = 3 independent experiments, analyzing a minimum of eight cells per condition). **c** Representative confocal images of primary rat cortical neurons infected with AAV expressing HA-tagged scrambled α-synuclein (AAV-Scr; top panel) or HA-tagged A53T α-synuclein (AAV-A53T; bottom panel). Nuclear counter staining with DAPI (blue), anti-HA (cyan), mCherry (red), and EGFP (green). Scale bar 10 μm (inset 5 μm). **d** Primary rat cortical neurons expressing A53T α-synuclein display an increase in mito-lysosomes (75.13 ± 8.79) in comparison to neurons expressing scrambled α-synuclein (56.47 ± 5.22) (one-tailed unpaired *T* test, *t*(81) = 1.85; *P* = 0.0336; *n* = 3 independent experiments, analyzing a minimum of 12 cells per condition). Data in graphs represent means ± s.e.m. ****P < 0.0001; *P < 0.05

We next examined mitophagy in a neuronal system as it has previously been shown that mitophagy can differ between cell types [21, 29]. To do this, we generated adeno-associated viruses (AAV) containing expression vectors of mito-QC (AAV-mito-QC) or human A53T α-synuclein (AAV-A53T α-synuclein) and co-transduced primary rat cortical neuron cultures. Cortical neurons tend to be affected during the later stages of PD when α-synuclein aggregation in these neurons is associated with cognitive impairment and dementia [30]. The transduction efficiency of the primary rat cortical neurons was approximately 50% which thereby allowed for the comparison between neurons transduced with AAV-A53T α-synuclein and those in the same culture without AAV-A53T α-synuclein as controls (Fig. 2a). Neurons with positive immunofluorescent staining for A53T α-synuclein had a significantly increased number of mito-lysosomes compared to A53T α-synuclein-negative neurons 96 h post-transduction (Fig. 2b). Next, we generated an AAV1 vector expressing a scrambled α-synuclein sequence (AAV-Scr) and similarly co-transduced primary rat cortical neuron cultures with AAV-mito-QC (Fig. 2c). Neurons overexpressing non-scrambled A53T α-synuclein exhibited an elevation in mito-lysosomes compared to AAV-Scr-positive neurons (Fig. 2d), suggesting that specific accumulation of mutant α-synuclein induces mitophagy.

### Blocking Lysosomal Function Suppresses Mutant α-Synuclein-Induced Mitophagy

**Fig. 3:**
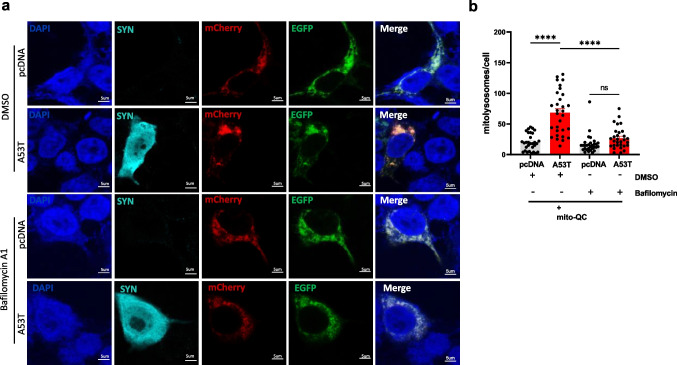
Blocking lysosomal acidification with bafilomycin A1 suppresses mutant α-synuclein-induced mitophagy. **a** Representative confocal images of SH-SY5Y cells transfected with mito-QC in combination with mutant A53T α-synuclein or pcDNA control and subsequently treated with bafilomycin A1 (40 nM) or DMSO (vehicle) for 6 h. Nuclear counter staining with DAPI (blue), anti-α-synuclein (cyan), mCherry (red), and EGFP (green). Scale bar 10 μm. **b** Cells expressing human A53T α-synuclein treated with DMSO exhibited an increase in the number of mito-lysosomes (68.2 ± 6.48), compared to pcDNA control cells treated with DMSO (20.17 ± 2.5) (one-way ANOVA, *F*(3,116) = 34.35; *P* < 0.0001; followed by Tukey’s m.c.t., *n* = 3 independent experiments, analyzing 10 cells per condition). Bafilomycin A1 treatment displays a significant decrease in the number of mito-lysosomes per cell (26.67 ± 3.2) compared to human A53T α-synuclein overexpressing cells treated with DMSO (68.2 ± 6.48). No differences in the levels of mito-lysosomes were observed between bafilomycin A1 and DMSO-treated pcDNA conditions. Data in the graph represents means ± s.e.m. ****P < 0.0001; ns indicates *P* > 0.05

We next assessed whether the quantifiable increase of the mCherry-only signal observed in mutant A53T α-synuclein conditions reflects increased mitochondrial turnover. To do so, we used bafilomycin A1, a selective vacuolar-type proton-pump inhibitor that prevents autophagy by blocking the acidification of lysosomes and suppresses mitophagy in PD models [31, 32]. Consistent with the data above, SH-SY5Y cells overexpressing human A53T α-synuclein exhibited elevated levels of mito-lysosomes compared to pcDNA-transfected cells similarly treated with vehicle, DMSO (Fig. 3a). However, bafilomycin A1 treatment led to a normalization of mito-lysosome levels in A53T α-synuclein overexpressing cells, comparable to pcDNA-transfected cells (Fig. 3b). Taken together, the results suggest that the accumulation of mitochondria within autophagosomes reflects increased mitochondrial turnover induced by mutant A53T α-synuclein.

### α-Synuclein-Mediated Mitophagy Dysfunction in the Substantia Nigra Precedes Dopaminergic Neuronal Loss In Vivo

**Fig. 4:**
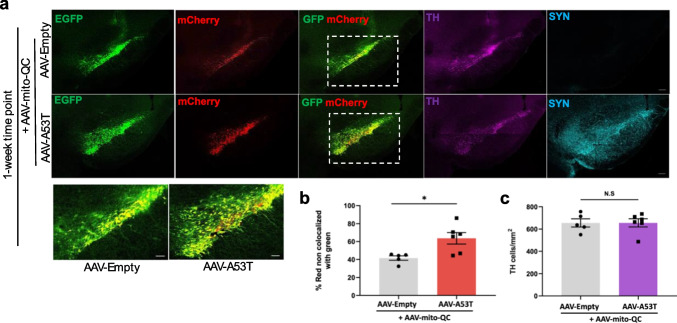
Mutant α-synuclein overexpression in rat SNpc is associated with increased levels of mitophagy at an early timepoint. **a** Representative confocal images of the SNpc in adult rats 1 week post-unilateral co-injections of AAV-mito-QC with either AAV-A53T α-synuclein or AAV-Empty control demonstrating fluorescent reporter mCherry (red), and EGFP (green), as well as immunofluorescent staining with anti-tyrosine hydroxylase (TH; magenta) and anti-α-synuclein (SYN; cyan). Scale bar 200 μm (inset 100 μm). **b** Quantification of red and green fluorescence signal within the SNpc reveals an increase in the red-only signal in the A53T α-synuclein condition in comparison to control, indicating an increase in mitophagy associated with A53T α-synuclein at 1 week post-injection (two-tailed unpaired *T* test, *t*(9) = 2.979; *P* = 0.0155; *n* = 5–6). Data is represented as mean ± s.e.m (*P* = 0.0155; unpaired *T* test; *n* = 5–6). **c** Quantification of TH + neurons per mm.^2^ within the SNpc reveals no difference in the density of nigral dopaminergic neurons 1 week post-AAV injections (two-tailed unpaired *T* test, *t*(9) = 0.0166; *P* = 0.9871; *n* = 5–6). Data in graphs represent means ± s.e.m. *P < 0.05; ns indicates *P* > 0.05

Based on our in vitro findings above, we then hypothesized that α-synuclein accumulation could also increase mitophagy in dopaminergic neurons of the SNpc, the primary neuroanatomical affected at the time of PD diagnosis. To test this hypothesis, we used a well-defined and characterized PD model in which α-synuclein expression is targeted to the SNpc by stereotactic injection of AAV-A53T α-synuclein. An advantage of this model over many of the transgenic mouse models is that there is progressive dopaminergic neurodegeneration due to α-synuclein which occurs over the course of weeks [22, 24–26]. To examine the effects of A53T α-synuclein overexpression on mitophagy in the rat SNpc prior to dopaminergic neuron loss, adult female Sprague–Dawley rats received co-injections of AAV-mito-QC with either AAV-A53T α-synuclein or AAV-Empty control, and we measured the mito-QC reporter signal within this midbrain region 1 week post-infection (Fig. 4a). We performed immunofluorescent staining for tyrosine hydroxylase (TH), a marker of dopaminergic neurons, and we found no difference in the number of TH-positive neurons between the two groups indicating no significant α-synuclein-mediated neuron loss at this timepoint. However, we found a significant increase in the red-only signal within the SNpc of animals co-injected with AAV-A53T α-synuclein compared to those co-injected with AAV-Empty control (Fig. 4b), which indicates an increase in mitophagy associated with A53T α-synuclein.

**Fig. 5:**
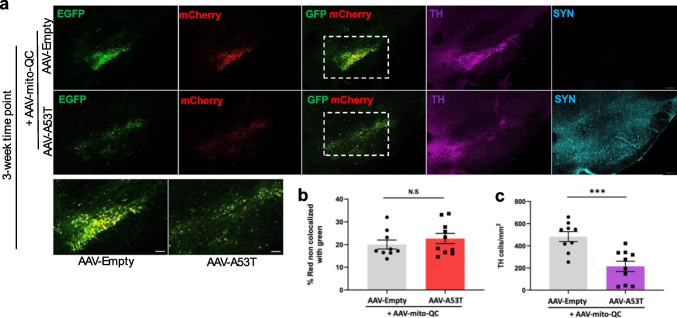
Mutant α-synuclein-mediated dopaminergic cell loss is associated with comparable levels of mitophagy at the 3-week timepoint. **a** Representative confocal images of the SNpc in adult rats 3 weeks post-unilateral co-injections of AAV-mito-QC with either AAV-A53T α-synuclein or AAV-Empty control demonstrating fluorescent reporter mCherry (red), and EGFP (green), as well as immunofluorescent staining with anti-tyrosine hydroxylase (TH; magenta) and anti-α-synuclein (SYN; cyan). Scale bar 200 μm (inset 100 μm). **b** Quantification of red and green fluorescence signal within the SNpc reveals no difference in the red-only signal in the A53T α-synuclein condition in comparison to control at 3 weeks post-injection (two-tailed unpaired *T* test, *t*(17) = 0.872; *P* = 0.3952; *n* = 9–10). **c** Quantification of TH + neurons per mm.^2^ within the SNpc reveals loss of nigral dopaminergic neurons 3 weeks post-A53T α-synuclein injection (two-tailed unpaired *T* test, *t*(17) = 4.177; *P* = 0.0006; *n* = 9–10). Data in graphs represent means ± s.e.m. ***P < 0.001; ns indicates *P* > 0.05

Finally, we studied a later timepoint using this rat model to define the temporal relationship between α-synuclein accumulation, mitophagy, and dopaminergic neurodegeneration in vivo. Adult female Sprague–Dawley rats received co-injections of AAV-mito-QC with either AAV-A53T α-synuclein or AAV-Empty control, and we measured the mito-QC reporter signal 3 weeks post-infection (Fig. 5a). At this later timepoint, there was a significant reduction in TH-positive cells within the SNpc in rats co-injected with AAV-A53T α-synuclein compared to those co-injected with AAV-Empty control. However, there was no difference in red-only signal within the SNpc between the two groups (Fig. 5b), indicating no differences in mitophagy at this timepoint. Taken together, our findings suggest that α-synuclein accumulation within dopaminergic neurons increases mitophagy at an early stage of the neurodegenerative process before loss of SNpc cells. At later stages, mitophagy returns to control levels, and this loss of enhanced mitophagy may contribute to dopaminergic neuron death.

## Discussion

Mounting evidence suggests that α-synuclein can adversely impact mitochondrial function and disrupt the mitochondria clearance process [11–16]. However, there exist discrepancies regarding whether α-synuclein causes increases or decreases in mitophagy in vitro. Additionally, there is limited in vivo data examining the effects of α-synuclein on mitophagy and determining whether it is an early or late event in the neurodegenerative process [11, 13]. In the present study, we demonstrate that overexpression of mutant A53T α-synuclein in both human SH-SY5Y cells and rat primary cortical neurons induces mitophagy. This increase in mitophagy was not observed in cells overexpressing WT α-synuclein, nor in cortical neurons transduced with a scrambled form of α-synuclein, but could be nullified by inhibition of lysosomal function. Furthermore, we identify early-onset mitophagy increases following the expression of A53T α-synuclein in dopaminergic neurons of the SNpc, which preceded neuronal loss. Later, mitophagy returned to control levels, potentially contributing to neurodegeneration due to the buildup of dysfunctional mitochondria in the cell. Taken together, our results suggest that mitochondrial dysfunction induced by mutant A53T α-synuclein is not a late-stage event in PD progression, but rather an early contributing factor driving the onset of disease.

Following the initial observation that α-synuclein can negatively impact mitochondrial function, several studies have demonstrated α-synuclein-mediated mitophagy dysregulation in PD pathogenesis. In vitro analyses have demonstrated both impaired and enhanced mitophagy associated with α-synuclein [12, 14–16]. This discrepancy may be in part due to different markers of varying specificity and sensitivity used to gauge mitophagy as well as the use of different cell models. To circumvent these potential problems, we utilized the binary-based fluorescence mito-QC mitophagy reporter [21], which co-localizes to the OMM and produces a readily quantifiable red signal when engulfed in an acidic lysosome. We found that mutant α-synuclein expression is associated with an increase in mito-lysosomes as measured by the mito-QC reporter as well as LC3-positive autophagosomes that co-localize with TOMM20-labeled mitochondria, which is in line with other studies demonstrating mitophagy induction [12, 15]. We further demonstrate that blocking lysosomal function inhibits mitophagy induced by mutant α-synuclein, indicating that the elevated levels of non-co-localized mCherry puncta observed are due to the increased engulfment of mitochondria by the lysosome for degradation. This is in line with a previous study which demonstrated that expression of A53T α-synuclein in rat primary cortical neurons results in the activation of macro-autophagy, which was associated with a global loss of mitochondria [12]. The congruity of these findings might partially be attributed to the consistent use of the same cell model, underscoring the importance of cell model consistency when drawing conclusions surrounding A53T α-synuclein-associated mitophagy.

To date, there have only been two in vivo studies that have looked at the effects of mutant α-synuclein on mitophagy, both of which used transgenic rodent models. The results from both studies similarly demonstrated that the expression of A53T α-synuclein leads to an increase in mitophagy within dopaminergic neurons [11, 13]. However, one study showed elevated mitophagy levels in 12-month-old A53T α-synuclein transgenic mice, while not reporting on dopaminergic cell loss [11], whereas the other reported elevated mitophagy markers in 3-week-old A53T α-synuclein transgenic mice and reported dopaminergic cell loss later on [13]. The differing timepoints used make it difficult to understand whether the effects of mutant α-synuclein on mitophagy are an early- or late-stage event in PD pathogenesis. In the latter study, A53T α-synuclein was associated with early-onset cytoplasmic inclusions containing mitochondria remnants, which occurred prior to dopaminergic cell death within the SNpc [13]. However, the observed cell death was delayed compared to the mitophagy defects, which was measured at 3 and 6 weeks, while significant neuronal loss in the SNpc did not occur until the 3-month mark [13]. This lapse of time between the two phenomena adds complexity when interpreting the relationship between mitophagy dysregulation and neurodegeneration. Our study demonstrates that A53T α-synuclein-mediated mitophagy dysregulations occur as early as 1 week post-synuclein expression, which preceded the neuronal loss within the SNpc observed 3 weeks post-expression. As it is often debated whether mitochondrial deficits in PD are the primary drivers of neurodegeneration, secondary effects, or part of a larger complex pathogenic process [33], the results here show that mitochondrial dysfunction may be an early event in synucleinopathy that drives neurodegeneration.

Numerous studies have highlighted that α-synuclein causes various forms of mitochondria damage, thereby necessitating the elimination of dysfunctional mitochondria [34]. In this study, the increased mitophagy observed within neurons of SNpc may be a compensatory mechanism to remove α-synuclein-mediated damaged mitochondria. By week 3, the marked increase in mitophagy was no longer observed, and at this timepoint, there was a clear loss of dopaminergic neurons within the SNpc of the rodent brains overexpressing A53T α-synuclein. We speculate that as time persists, the buffering capacity of the mitophagy process is overridden by the deteriorating cellular environment, resulting in a buildup of defective mitochondria and leading to cell death. The finding that α-synuclein induces an initial, transient, increase in mitophagy that subsequently reduces to control levels may explain some of the discrepancies in the literature regarding the direction by which α-synuclein affects mitochondrial clearance. Alternatively, mutant α-synuclein could also impact the mitochondria by improperly promoting the removal of the healthy organelle [12, 13], resulting in bioenergetic defects for the cell. It is also possible that α-synuclein is affecting other pathways, which indirectly impacts autophagy pathways. It is well established that both the ubiquitin–proteasome pathway (UPS) and autophagy-lysosome pathway (ALP) play important roles in PD, as these two clearance pathways are important for protecting against α-synuclein toxicity [35]. Employing the same animal model used in this study, we have previously shown that A53T α-synuclein causes early-onset impairments of the UPS in dopaminergic neurons, which precedes neurodegeneration within the SNpc [25]. Therefore, we may also speculate that mutant α-synuclein negatively impacts the UPS, which subsequently upregulates autophagy [36], leading to the excessive removal of mitochondria, promoting bioenergetic defects within the cell, and contributing to cell death.

While this study demonstrates early dysregulation of mitophagy precedes dopaminergic cell death, it does not address why there is an increase in mitochondrial clearance associated with α-synuclein. We also do not compare the relative effects that mutant α-synuclein has on the ALP versus other clearance pathways such as the UPS. Since we have previously found that UPS dysfunction also precedes neuronal loss in the presence of A53T α-synuclein [25], it is likely that both the UPS and ALP are important therapeutic targets for reducing the detrimental impact of α-synuclein on dopaminergic neurons. Understanding the mechanism(s) that promote early-onset mitophagy will be a critical step moving forward in the AAV-based α-synuclein model of PD. Since AAV-mediated expression of α-synuclein induces an acute and severe loss of dopaminergic neurons in this model [37], it will be important to elucidate whether mitophagy further declines during late-stage pathology and how this correlates with more gradual cell loss in transgenic mice expressing A53T.

In summary, we have identified that the expression of mutant α-synuclein results in early dysregulations in mitophagy which precedes dopaminergic neuron degeneration within the SNpc of rodent brains. This was readily observed using the mito-QC mitophagy reporter and highlights that α-synuclein-induced mitochondrial dysfunction may be an early event in PD pathology driving dopaminergic neuronal degeneration. Limiting the interaction between mutant α-synuclein and the mitochondria may be a promising therapeutic target for the development of disease-modifying treatments in PD.

## Supplementary Information

Below is the link to the electronic supplementary material.

Supplementary file1 (DOCX 16 KB)

## Data Availability

All data generated or analyzed during this study are included in this published article (and its supplementary information files).
