# Supplementary material for: Mitophagy Upregulation Occurs Early in the Neurodegenerative Process Mediated by α-Synuclein
Source: Mol Neurobiol. 2024 Apr 6;61(11):9032–42. doi: 10.1007/s12035-024-04131-6 (PMC11496319; doi:10.1007/s12035-024-04131-6)
Supplement: Supplementary file 1 — Supplementary file1 (DOCX 16 KB) [file 12035_2024_4131_MOESM1_ESM.docx]

**Table S1** Primary antibodies used for immunofluorescent staining of cryosections.

| **Antibody** | **Dilution** | **Supplier** |
| --- | --- | --- |
| Anti-alpha synuclein | 1: 500 | ThermoFisher Scientific (32-8100) |
| Anti-tyrosine hydroxylase | 1:1000 | ThermoFisher Scientific (PA585167) |

**Table S2** Secondary antibodies used for immunofluorescent staining of cryosections.

| **Antibody** | **Dilution** | **Supplier** |
| --- | --- | --- |
| AlexaFluor®405 Goat anti-rabbit IgG | 1:500 | Invitrogen (A-31556) |
| AlexaFluor®647 Goat anti-mouse IgG | 1:500 | Invitrogen (A-21235) |
